# 

**Published:** 1889-07

**Authors:** 


					﻿JULY, 1889.
OLD SERIES
Vol. XXV
NEW SERIESI
Vol. VI.-No.
& 1855 O

■wMsum
JOURNAL

^3Tt^A^V>Tjf

W. F. WESTMORELAND, M.D?
H. V. M. MILLER, M. D. LL. D.<
VIRGIL 0. HARDON, M. D.
PUBLISHED BY JAMES P.HARRISON&C$
________________«flTLANTA,GA. M
SUBSCRIPTION t S2.6O A YEAR-
				

